# Exclusive waterpipe smoking and the risk of nasopharynx cancer in Vietnamese men, a prospective cohort study

**DOI:** 10.1038/s41598-023-40253-y

**Published:** 2023-08-14

**Authors:** Thinh Gia Nguyen, Hung Dinh Kieu, Dung Thuy Thi Truong, Khoa Xuan Ngo, Shunya Ikeda, Ngoan Tran Le

**Affiliations:** 1 School of Medicine, International University of Health and Welfare, Narita, Japan; 2 Department of Surgery, Hanoi Medical University, Hanoi, Vietnam; 3 University of Medicine and Pharmacy at Ho Chi Minh City, Ho Chi Minh City, Vietnam; 4 Graduate School of Public Health, International University of Health and Welfare, Narita, Japan; 5 Department of Anatomy, Hanoi Medical University, Hanoi, Vietnam; 6 Institute of Research and Development, Duy Tan University, Da Nang, Vietnam; 7 Department of Occupational Health, Institute for Preventive Medicine and Public Health, Hanoi Medical University, Hanoi, Vietnam

**Keywords:** Cancer, Public health, Epidemiology, Cancer, Risk factors

## Abstract

Tobacco smoking is carcinogenic to humans. Besides cigarettes, the most common form of tobacco smoking, there was sparse evidence of waterpipe’s carcinogenicity-induced nasopharyngeal cancer (NPC). This study investigated the association between waterpipe smoking and NPC mortality. Our study followed up with 20,144 eligible man participants from nine northern Vietnam communes between 2007 and 2019. Face-to-face interviews were conducted to gather data on exclusive waterpipe and cigarette smoking and dietary intake using structured semi-quantitative food frequency and lifestyle questionnaires. Nasopharyngeal cancer was determined by accessing the medical records at the state health facilities. We estimated the Cox proportional hazard ratio and 95% confidence intervals, HR (95% CI). The proportion of never smokers, exclusive waterpipe, exclusive cigarette, and dual waterpipe and cigarette smokers was 55.8%, 14.5%, 16.6%, and 13.1%, respectively. Exclusively waterpipe smokers increased the risk of NPC death compared to exclusively cigarette smokers, HR (95% CI): 4.51 (1.25, 16.31), *p* = 0.022. A dose-dependent positive relationship between NPC and exclusive waterpipe smoking was significantly seen for higher intensity HR (95% CI): 1.35 (1.07, 1.71), earlier age of smoking initiation HR (95% CI): 1.26 (1.06, 1.50), longer duration HR (95% CI): 1.31 (1.04, 1.66), and the cumulative number of a smoke lifetime HR (95% CI): 1.37 (1.08, 1.74). We observed a significant positive association between exclusive waterpipe smoking and NPC in men. The findings suggested that waterpipe smoking is likely more harmful than cigarettes in developing this cancer. A firm tobacco control against waterpipe smoking is highly recommended.

## Introduction

Nasopharyngeal cancer (NPC) is rare worldwide, with an incident rate of under 1 per 100,000 population per year^1^. The incidence rate in men is double or triple that in women^1^. However, in the Cantonese population of southern China, substantially higher rates are reported^1^. Intermediate rates are found in indigenous people in Southeast Asia, the Arctic region, North Africa, and the Middle East^1^. In 2018, 85% of an estimated 129,079 incident cases of NPC were diagnosed in Asia^1^. In Southeast Asia, NPC ranked 9th among incident cancers and 8th among cancer deaths^1^. In China, the highest-risk population belongs to the southeastern Chinese province of Guangdong, the Cantonese-speaking population^2^. In Southeast Asia, NPC risk appears higher in the Chinese^3^. In the United States and Singapore, rates are highest among the Chinese^1^. NPC ranked 9th most common cancer in Vietnam, with 6040 new cases in 2020^4^. There is an unclear association between race and ethnicity and NPC incidence.

NPC is classified histologically as keratinizing squamous cell carcinoma (type I); differentiated nonkeratinizing carcinoma (type II); undifferentiated nonkeratinizing carcinoma (type III); or basaloid squamous cell carcinoma, a rare subtype^5^. Undifferentiated carcinoma (type III) comprises over 95% of the NPC cases in high-incidence regions, while differentiated carcinoma (type I) is predominant in low-incidence areas^6^.

Risk factors of NPC updated in recent studies include Cantonese ethnicity, man sex, Epstein–Barr virus (EBV) infection, a family history of NPC, high consumption of salt-preserved fish, low intake of fresh vegetables and fruits intake, smoking, and some human leukocyte antigen (HLA) class I alleles^7^.

IARC Working Groups reported sufficient evidence in humans for the carcinogenicity of tobacco smoking^8^. Tobacco smoking is classified into group 1 carcinogens to humans. Substantial evidence showed a causal relationship between tobacco smoking and cancers of the lung^9^ and oral cavity^10^. Cohort studies reported a positive association between cigarette smoking and NPC in the U.S., Taiwan, and Singapore but inconclusion in the U.K. populations^11–15^. However, waterpipe tobacco smoking was not examined in these studies.

Besides cigarette smoking, waterpipe is an older form of tobacco smoking. Among men, Vietnam has the highest rate of waterpipe tobacco smoking, followed by China and Malaysia^16^. Vietnamese waterpipe is made of bamboo, metal, or porcelain with structures and directions similar to the ones used in China^16^. Waterpipe smoking in Vietnam is more prevalent in the older, rural, less educated man population^16^. According to the National Health Survey in Vietnam, the prevalence of man smokers was 51.2%. Among man smokers, most of them smoked cigarettes only (69.1%), followed by Vietnamese WPT only (23.2%) and both products (7.7%) in 2001–2002^17^.

Evidence of the relationship between cigarette and waterpipe smoking and NPC is generally limited. Thus, our study aimed to investigate the relationship between cigarette and waterpipe smoking and NPC mortality after adjusting for confounding factors.

## Methods

### Study design and population

**Figure 1:**
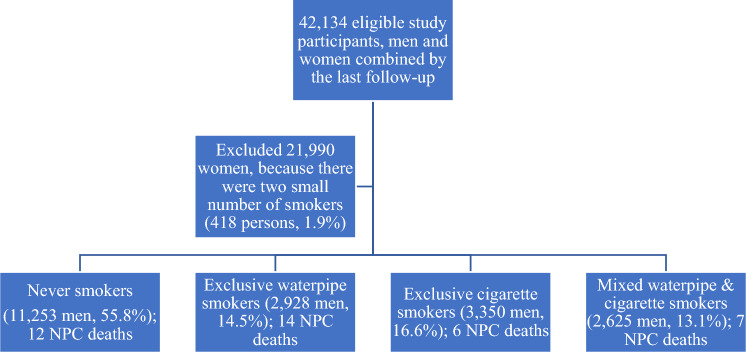
Eligible study participants in 20,144 men by smoking status. *NPC* nasopharyngeal cancer.

In 2007, 52,325 individuals from 12,746 households were recruited into our prospective cohort study. They belonged to nine communes in Hung Yen, Phu Pho, and Hanoi in Northern Vietnam. A questionnaire on exclusive waterpipe and cigarette smoking, demographic characteristics, dietary intake, fridge availability, cooking methods, and alcohol consumption was used to ask the participants. Then, we followed up on all the causes of death using medical records collected at the state health facilities. Deaths caused by cancer were coded based on ICD-10. Participants with (1) no history of cancer and (2) presence at the research location through the investigation period were included in our prospective cohort study. After over 12 years of follow-up, 7182 participants aged under 10-year-old and loosed follow-up 2997 persons due to migration were excluded. We excluded 21,990 women because there were two small numbers of smokers (418 persons, 1.9%), Fig. 1. Finally, the data of 20,144-man participants with 39 NPC mortality cases were examined in the present study.

### Exposure assessment

Trained students of Hanoi Medical University conducted face-to-face interviews with participants. We used a structured questionnaire to obtain information about tobacco consumption in the participants who have already smoked at least one waterpipe tobacco and cigarette entirely in their lifetime.

Smoking status was categorized into three groups: (1) never-smokers: participants who never smoked any cigarette or waterpipe during their lifetime; (2) former smokers: participants who smoked a cigarette or waterpipe in the past but had quit at the time of the interview; and (3) current smokers: participants who currently smoked cigarette or waterpipe at the time of the interview.

Our study also collected smoking data on intensity, duration, and ages at started smoking. Both current and ex-smokers were asked how many smokes per day. The number of smokes was categorized into never, less than 21 per day, and over 20 per day. For participants who didn’t smoke daily but occasionally during the week, the average daily smoking, calculated by the total amount of tobacco in 1 week divided by 7 days, was used. Duration of smoking (in years) was classified into never, less than 21 years, and over 20 years. Age at started smoking was categorized into never, over 20 years old, and less than 21 years old. The cumulative number of a smoke lifetime was calculated by average daily smoking (365 days) multiplied by the duration of smoking (year). This index was categorized into never, less than 201 times, and over 200 times.

### Outcome ascertainment

The permanently appointed health workers collected NPC mortality and other causes of death monthly, including a medical doctor at each state commune health station. The underlying causes of death, the immediate cause of death, and contributing cause of death were determined by linking medical records available at the state commune health station (CHS), district hospital, provincial hospital, and other health facilities, or death certificates issued by the hospital where the patients had died. Within the first month of the death, the CHS of each commune in Vietnam received reports of all death cases from each family and clarified the underlying cause of death. Then, the CHS coded the cause of death according to ICD-10^18^. The malignant neoplasm of the nasopharynx was coded as C11^18^. In cases of dying at home without medical records in any hospital, validated WHO verbal autopsies were applied to determine the cause of death. A verbal autopsy is an interview with family members and caregivers of the deceased using a structured questionnaire to elicit signs and symptoms and other important information that can be used to determine the most likely cause of death^19^. Staff involved in the study followed the coding guidelines to apply ICD-10 rules to the diagnoses resulting from such an autopsy^19^. In populations lacking vital registration and medical certification, including Vietnam, this technique has become the primary source of information about causes of death^19^. Additionally, it was reported that verbal autopsies' sensitivity and positive predictive value reached 75 to 100% in the Vietnamese population^20^.

### Follow-up and censoring of study participants

The follow-up of our study ended on December 31, 2019. Follow-up time in person-years was calculated from the baseline to the date of death from any cause, including NPC, the date the participant left the study areas, or the end of the follow-up period, whichever came first. In the current study, 217,531 person-years were estimated as the total for 20,144 men.

### Covariate information

Our study adjusted for potential confounding factors suggested in the previous studies^6,7^. Covariates in this study include age; sex; education level; fridge availability at the household (represented socio-economic status); body mass index (BMI); alcohol consumption; total energy (kcal/day), protein (g/day), lipid (g/day), and carbohydrate (g/day) intake. The age (years) of the study population was categorized as follows (10–19, 20–29, 30–39, 40–49, 50–59, 60–69, 70–79, and 80+). Education level was classified into three groups: < 6 years (primary school or lower), 7+ years (secondary school or higher), and unknown. According to the recommendation of the World Health Organization for the Asian population, BMI was categorized into underweight (< 18.5 kg/m^2^), average (18.5–< 23 kg/m^2^), and overweight (23+ kg/m^2^)^21^. Fridge availability was either a “yes” or “no.” This index also indicated the participants’ economic status. Alcohol consumption was also classified as “yes” (ever consumed alcoholic drinks) or “no” (never consumed alcoholic beverages).

A validated semi-quantitative food frequency questionnaire (SQFFQ) was applied to the face-to-face interview to assess each food item's consumption frequency (using a specific portion size) over the last 12 months. SQFFQ was validated for evaluating nutrient intake in the general population in Northern Vietnam in 2017^22^. Frequency of information was categorized into nine groups: never or < 1/month, 1–3/month, 1/week, 2–4/week, 5–6/week, 1/day, 2–3/day, 4–5/day, and ≥ 6/day. The Vietnam Food Composition Table was used to compute the nutrient composition^23^. Nutrient intake, including total energy, protein, lipid, and carbohydrate, was calculated by multiplying the nutrient composition of foods by the average daily intake and the reported frequency per year^24^.

### Statistical analysis

Cox proportional hazard regression analysis was used to calculate hazard ratios (HR) and corresponding 95% confidence intervals (95% CI) to assess the association between exclusive waterpipe, exclusive cigarette, and NPC mortality. Never Smokers was used as a reference group. We used the Kaplan–Meier method to determine the survival estimates of waterpipe smoking compared to exclusive cigarette smoking. All tests were two-sided, and a *p*-value of less than 0.05 was accepted as statistically significant. In multivariable-adjusted models, the dose–response relationship was investigated using the trend test.

### Ethics approval and consent to participate

The authors confirm following the study protocol that was approved by the Ethics Committee of IRB-Hanoi Medical University, Vietnam, for ethics in biomedical research implementation (Approval number NCS33/HMU-IRB dated 29 March 2019) and the IRB-International University of Health and Welfare, Japan (Approval number 21-Ig-92 dated 21 August 2021). The study is performed without intervention, a secondary analysis using existing data. We used the method of anonymous. Data is saved into a USB and private computer hard disk with a password. The principal investigator keeps the USB and computer secure and will not allow others to go through them except research team members. The data will be saved for 10 years after publication.

All methods were performed and carried out following relevant ethical guidelines and Vietnam's national regulations. We obtained written informed consent from all 12,746 households and their family members of 52,325 participants. All answers about smoking habits, diet-related factors, and family history will be anonymous by numbers.

## Results

**Figure 2:**
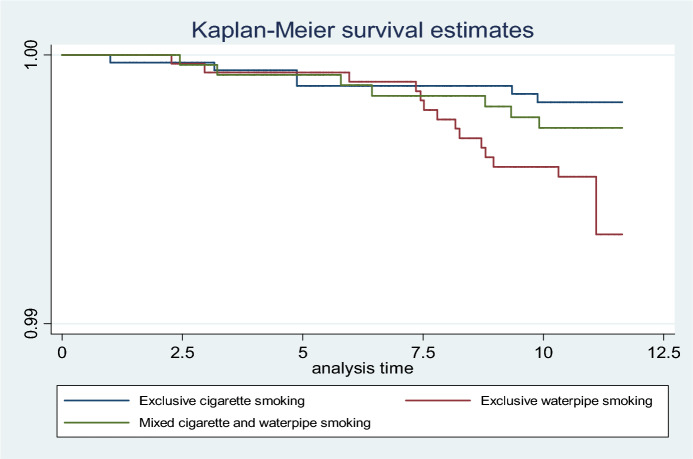
Kaplan–Meier survival estimates for the exclusive cigarette, exclusive waterpipe, and mixed cigarette and waterpipe smoking.

Among 20,144-man participants, the proportion of never smokers, exclusive waterpipe smokers, exclusive cigarette smokers, and dual waterpipe and cigarette smokers was 55.8%, 14.5%, 16.6%, and 13.1%, respectively. NPC death was 39-man cases and seven-woman cases. The proportion of women smokers was 1.9%, and they were excluded from the final analysis, Fig. 1. Survival estimates among exclusive waterpipe smokers were lowest compared to those of exclusive cigarette smokers. Dual cigarette and waterpipe smokers had an intermediate risk of decreased survival estimates, Fig. 2.

A null association between dual cigarette and waterpipe smoking and NPC mortality was seen according to intensity and years of smoking. Smoking before 21 significantly increased the risk of NPC, multivariable-HR (95% CI): 5.07 (1.57, 16.39), Table 1. A null association between exclusive cigarette smoking and NPC mortality was observed by the exposure to smoking intensity, duration, age of smoking initiation, and cumulative number of smoking lifetime, Table 2. A strong positive association between exclusive waterpipe smoking and NPC mortality according to intensity, duration, age of smoking initiation, and cumulative number of smoking lifetime. Per increment level of these exposures, respectively, multivariable-HR (95% CI): 1.35 (1.07, 1.71), *p* = 0.011; Multivariable-HR (95% CI): 1.26 (1.06, 1.50), *p* = 0.011; Multivariable-HR (95% CI): 1.31 (1.04, 1.66), *p* = 0.022 and Multivariable-HR (95% CI): 1.37 (1.08, 1.74), *p* = 0.011 were estimated, Table 3. Compared to exclusive cigarette smokers, exclusive waterpipe smokers significantly elevated the risk of NPC, multivariable-HR (95% CI): 4.51 (1.25, 16.31), *p* = 0.022. Dual cigarette and waterpipe smokers also increased the risk of NPC but insignificant, multivariable-HR (95% CI): 2.66 (0.66, 10.70), *p* = 0.167, Table 4.

**Table 1:** Dual cigarette and waterpipe smoking and the risk of nasopharynx cancer in Vietnamese men.

Smoking status	Person-year	Cases	Age-adjusted HR (95% CI)	*p*	Multivariable -adjusted HR (95% CI) *	*p*
Number of smokes per day						
Never smoking	122,453	12	1.00		1.00	
Less than 21	17,851	4	1.57 (0.51, 4.88)	0.432	1.78 (0.56, 5.65)	0.324
Over 20	9828	3	1.73 (0.49, 6.14)	0.396	2.11 (0.58, 7.71)	0.257
Per increment level of exposures	150,132	19	1.15 (0.85, 1.56)	0.364	1.21 (0.89, 1.65)	0.229
Ages at started smoking						
Never smoking	122,453	12	1.00		1.00	
Over 20	16,960	3	1.15 (0.32, 4.06)	0.833	1.36 (0.37, 4.97)	0.643
Less than 21	9002	4	3.93 (1.26, 12.27)	0.018	5.07 (1.57, 16.39)	0.007
Per increment level of exposures	148,415	19	1.19 (0.96, 1.47)	0.109	1.25 (1.00, 1.56)	0.050
Years of smoking						
Never smoking	122,453	12	1.00		1.00	
Less than 21	20,124	3	1.26 (0.35, 4.46)	0.725	1.56 (0.42, 5.72)	0.505
Over 20	6730	4	2.29 (0.72, 7.22)	0.158	2.72 (0.83, 8.89)	0.097
Per increment level of exposures	149,307	19	1.23 (0.92, 1.64)	0.158	1.28 (0.96, 1.72)	0.093

*HR (95% CI)*, azard ratio (95% confidence intervals); *Adjusted for age groups (10–19, 20–29, 30–39, 40–49, 50–59, 60–69, 70–79, 80+), sex, education level (< 6 years, 7+ years, unknown), available fridge (yes/no, unknown), BMI (kg/m^2^, < 18.5, 18.5–< 23, 23+, unknown), alcohol consumption (yes/no, unknown), total energy intake (kcal/day, quintiles), protein intake (g/day, quintiles), lipid intake (g/day, quintiles), carbohydrate intake (g/day, quintiles).

**Table 2:** Exclusive cigarette smoking and the risk of nasopharynx cancer in Vietnamese men.

Smoking status	Person-year	Cases	Age-adjusted HR (95% CI)	*p*	Multivariable -adjusted HR (95% CI) *	*p*
Number of smokes per day						
Never smoking	122,453	12	1.00		1.00	
Less than 13	26,063	4	1.01 (0.33, 3.13)	0.986	1.03 (0.33, 3.24)	0.954
Over 12	9240	2	1.14 (0.25, 5.10)	0.865	1.11 (0.25, 5.03)	0.891
Per increment level of exposures	157,757	18	1.03 (0.71, 1.50)	0.868	1.03 (0.71, 1.49)	0.889
Ages at started smoking (missing 3 cases)						
Never smoking	122,453	12	1.00		1.00	
Over 29	7006	2	1.43 (0.32, 6.41)	0.639	1.44 (0.32, 6.55)	0.636
Less than 30	22,239	1	0.39 (0.05, 3.01)	0.366	0.36 (0.05, 2.83)	0.333
Per increment level of exposures	151,699	15	0.93 (0.70, 1.22)	0.587	0.92 (0.69, 1.21)	0.542
Years of smoking						
Never smoking	122,453	12	1.00		1.00	
Less than 11	19,829	3	1.38 (0.39, 4.90)	0.622	1.45 (0.40, 5.23)	0.567
Over 10	15,157	3	0.85 (0.24, 3.04)	0.805	0.83 (0.23, 3.01)	0.782
Per increment level of exposures	157,439	18	0.97 (0.71, 1.31)	0.822	0.96 (0.70, 1.31)	0.798
Cumulative number of smokes lifetimes						
Never smoking	122,453	12	1.00		1.00	
Less than 201	26,878	4	1.08 (0.35, 3.35)	0.894	1.12 (0.36, 3.51)	0.847
Over 200	7443	2	1.06 (0.24, 4.79)	0.936	1.01 (0.22, 4.64)	0.986
Per increment level of exposures	156,775	18	1.02 (0.70, 1.47)	0.922	1.01 (0.70, 1.46)	0.965

*HR (95% CI)*, azard ratio (95% confidence intervals); *Adjusted for age groups (10–19, 20–29, 30–39, 40–49, 50–59, 60–69, 70–79, 80+), sex, education level (< 6 years, 7+ years, unknown), available fridge (yes/no, unknown), BMI (kg/m^2^, < 18.5, 18.5–< 23, 23+, unknown), alcohol consumption (yes/no, unknown), total energy intake (kcal/day, quintiles), protein intake (g/day, quintiles), lipid intake (g/day, quintiles), carbohydrate intake (g/day, quintiles).

**Table 3:** Exclusive waterpipe smoking and the risk of nasopharynx cancer in Vietnamese men.

Smoking status	Person-year	Cases	Age-adjusted HR (95% CI)	*p*	Multivariable -adjusted HR (95% CI) *	*p*
Number of smokes per day						
Never smoking	122,453	12	1.00		1.00	
Less than 11	19,066	8	2.26 (0.92, 5.57)	0.076	2.47 (0.99, 6.15)	0.053
Over 10	10,396	6	3.11 (1.16, 8.32)	0.024	3.58 (1.32, 9.71)	0.012
Per increment level of exposures	151,915	26	1.31 (1.04, 1.65)	0.022	1.35 (1.07, 1.71)	0.011
Ages at started smoking (missing 2 cases)						
Never smoking	122,453	12	1.00		1.00	
Over 25	12,038	7	3.17 (1.24, 8.06)	0.016	3.37 (1.31, 8.67)	0.012
Less than 26	14,387	5	2.21 (0.78, 6.29)	0.136	2.58 (0.90, 7.42)	0.079
Per increment level of exposures	148,879	24	1.23 (1.03, 1.46)	0.021	1.26 (1.06, 1.50)	0.011
Years of smoking (missing 1 case)						
Never smoking	122,453	12	1.00		1.00	
Less than 26 years	20,789	6	2.13 (0.80, 5.69)	0.130	2.39 (0.89, 6.45)	0.084
Over 25 years	7839	7	2.83 (1.06, 7.50)	0.037	3.10 (1.16, 8.26)	0.024
Per increment level of exposures	151,081	25	1.29 (1.02, 1.63)	0.035	1.31 (1.04, 1.66)	0.022
Cumulative number of smokes lifetimes (missing 1 case)						
Never smoking	122,453	12	1.00		1.00	
Less than 301	21,333	7	2.05 (0.81, 5.21)	0.132	2.30 (0.89, 5.90)	0.084
Over 300	7030	6	3.31 (1.22, 9.03)	0.019	3.65 (1.33, 10.06)	0.012
Per increment level of exposures	150,816	25	1.34 (1.05, 1.70)	0.017	1.37 (1.08, 1.74)	0.011

*HR (95% CI)* hazard ratio (95% confidence intervals); *Adjusted for age groups (10–19, 20–29, 30–39, 40–49, 50–59, 60–69, 70–79, 80+), sex, education level (< 6 years, 7+ years, unknown), available fridge (yes/no, unknown), BMI (kg/m^2^, < 18.5, 18.5–< 23, 23+, unknown), alcohol consumption (yes/no, unknown), total energy intake (kcal/day, quintiles), protein intake (g/day, quintiles), lipid intake (g/day, quintiles), carbohydrate intake (g/day, quintiles).

**Table 4:** Exclusive waterpipe smoking compared to exclusive cigarette smoking and the risk of nasopharynx cancer in Vietnamese men.

Smoking status	Person-year	Cases	Age-adjusted HR (95% CI)	*p*	Multivariable -adjusted HR (95% CI)*	*p*
Exclusive cigarette	36,327	6	1.00		1.00	
Exclusive waterpipe	30,957	14	2.43 (0.93, 6.34)	0.069	4.51 (1.25, 16.31)	0.022
Dual cigarette and waterpipe	27,794	7	1.61 (0.54, 4.79)	0.394	2.66 (0.66, 10.70)	0.167

*HR (95% CI)* hazard ratio (95% confidence intervals); *Adjusted for age groups (10–19, 20–29, 30–39, 40–49, 50–59, 60–69, 70–79, 80+), sex, education level (< 6 years, 7+ years, unknown), available fridge (yes/no, unknown), BMI (kg/m^2^, < 18.5, 18.5–< 23, 23+, unknown), alcohol consumption (yes/no, unknown), total energy intake (kcal/day, quintiles), protein intake (g/day, quintiles), lipid intake (g/day, quintiles), carbohydrate intake (g/day, quintiles) and age at started of smoking, average number of daily smoking.

## Discussion

We observed a decrease in survival estimates and a significantly higher mortality rate of NPC among exclusive waterpipe smokers than exclusive cigarette smokers. Compared to never smoking, a strong positive association between exclusive waterpipe smoking and NPC mortality according to intensity, duration, age of smoking initiation, and cumulative number of smoking lifetime was also reported.

The association between cigarette smoking and NPC mortality remains limited. Friborg et al. conducted a prospective study on Singapore Chinese, the high-risk population that comprised the majority of undifferentiated NPCs (nearly 90%)^14^. This cohort study also did not report a statistically increased risk of NPC among current cigarette smokers compared with never smokers. The difference in the effect of smoking on NPC risk might depend on the histological type of NPC. In a recent study, undifferentiated carcinoma, which is the most common type of NPC in high-risk areas, seemed more strongly related to EBV infection other than cigarette smoking^25^. A lot of viral oncogenes related to EBV have been documented, such as Epstein–Barr nuclear antigen 1 (EBNA1), EBV-encoded small RNA 1/2 (EBER1/2), and Latent membrane protein 1 (LMP1)^25^. On the other hand, the hazard risk of cigarette smoking was found to be higher for differentiated than undifferentiated NPC^26–28^. Unlike undifferentiated NPC, differentiated NPC only accounts for a minor portion of NPC cases in such a high-risk population as Vietnam^6^. Wan-Lun Hsu et al. reported that EBV and cigarette smoking correlate with NPC^25^. In experimental studies, to develop nasopharyngeal carcinoma, EBV latent infection needs to be established on genetically altered or inflammatory nasopharyngeal epithelial cells^15,29^. Smoking may initiate the EBV-mediated carcinogenic process by damaging DNA through many pathways similar to smoking-induced lung cancer^8^. Mutations in KRAS and TP53 are well-known in smoking-related lung cancers^8^.

To the best knowledge, our study is the only one that reported the association between exclusive waterpipe and NPC. Regarding a similar Arabian waterpipe called shisha, there is only one case–control analysis of the relationship between shisha smoking and NPC. Feng et al. reported a null association between Shisha smoking and NPC with an odds ratio of 0.49 (95% CI 0.20–1.23)^30^. This case–control study might be suffered from a recall bias when the proportion of ever Shisha smoking was only 2% (9/450 NPC cases).

Waterpipe smoking was reported to be associated with oral cancer, oesophageal cancer, urinary bladder cancer, especially respiratory diseases, and lung cancer, regarding long-term health hazards^31^. There is evidence of the association between waterpipe smoking and chronic obstructive pulmonary disease^32^. This disease is strongly associated with lung cancer. Other head and neck cancers, such as oral cancer, are reported to have a positive association with waterpipe smoking^33,34^.

Waterpipe is believed to be safer than cigarette smoking for a long time because the smoke is filtered through a column of water before being inhaled. However, even passing through water, the eliminated concentration of toxins is limited. It is reported that a single waterpipe session exposes the smoker to 3–9 times the CO, 1.7 times the nicotine, 8–15 times PAHs, 6–9 times formaldehyde, as well as heavy metals such as Arsenic, Chromium, and Lead when compared to cigarette smoking^35^. Moreover, waterpipe tobacco involves Nicotiana rustica leaves that contain a higher level of nicotine (9%) than cigarettes (1–3%)^36^. Additionally, tobacco is indirectly heated at a lower temperature. These toxic substances produced by waterpipe smoking may be different from or even more hazardous than cigarettes.

There is no precise mechanism explaining the relationship between waterpipe and NPC. Over 60 carcinogens can covalently bind to DNA directly or after metabolism, forming DNA adducts^8^. DNA adducts are central to chemical carcinogenesis because they can cause miscoding and permanent mutations^8^. Aside from this significant pathway, though not carcinogenic, high exposure to nicotine in waterpipe smokers may facilitate carcinogenesis by activating serine/threonine kinase Akt (also known as protein kinase B), protein kinase A, and other changes^8^. PAHs and nitrosamines may account for some base-substitution mutagenicity^8^. In addition, waterpipe smoking increased transmittable risk due to the shared pipe among smokers. Waterpipe-mediated EBV infection has been cautioned by WHO^37^. Again, EBV infection is an important risk of NPC^25^. These factors suggest that waterpipes may be more hazardous to health than cigarettes.

This study has many strengths. First, this is a prospective cohort study performed in a high-risk population. The subgroup analyses of various smoking habits, such as smoking status, intensity, duration, age of smoking initiation, and the cumulative amount of smoking, produced a precise quantification of the relationship between tobacco and NPC mortality. Exclusive cigarette, exclusive waterpipe, and dual cigarette and waterpipe smoking were stratified to estimate how the mortality hazard rate differs by smoking type. Second, advanced adjustment variables for age, sex, education level, available fridge, BMI, alcohol consumption, total energy intake, protein intake, lipid intake, and carbohydrate intake were included.

Our study has certain limitations. First, histological types of NPC cases and EBV infection status were not assessed. Further studies should investigate the relationship between cigarette and waterpipe smoking, EBV, and NPC. Second, our study analyses Vietnamese men in the high-risk population only. Therefore, our findings may not be generalized to women and people not in endemic regions. Third, although the general sample of this cohort is large, the NPC death cases are limited. This is an attribute of the low incidence of this type of cancer. Further investigations, such as case–control studies and review papers, are needed to enhance the evidence of this association. Lastly, mortality was used as an outcome determination, which may underestimate the association between smoking and NPC.

## Data Availability

The datasets used and analyzed during the current study are available from the corresponding author upon reasonable request.

## Abbreviations

NPC: Nasopharyngeal cancer
BMI: Body mass index
OR (95% CI): Odds ratio (95% confidence interval)
SQFFQ: Semi-quantitative food frequency questionnaire
EBV: Epstein–Barr virus
CHS: Commune health station
